# Perceived Suitability for Being a Dental Hygienist and Self‐Compassion: A 6‐Month Longitudinal Cohort Study

**DOI:** 10.1002/jdd.70174

**Published:** 2026-02-04

**Authors:** Maya Izumi, Hsiu‐Yueh Liu, Sumio Akifusa

**Affiliations:** ^1^ School of Oral Health Sciences Faculty of Dentistry Kyushu Dental University Fukuoka Japan; ^2^ Department of Oral Hygiene College of Dental Medicine Kaohsiung Medical University Kaohsiung Taiwan

**Keywords:** dental hygiene students, longitudinal study, perceived professional suitability, self‐compassion

## Abstract

**Objectives:**

Perceived professional suitability—students' judgment of whether they are well‐suited to their profession—may shift within a semester, yet longitudinal evidence in dental hygiene education is limited. We examined 6‐month changes in perceived suitability and their psychological associations.

**Methods:**

This longitudinal survey followed dental hygiene students from April to September 2025 in Kitakyushu, Japan. Of 450 eligible students, 402 responded at baseline, and 293 completed both time points. The primary outcome at 6 months was perceived suitability for being a dental hygienist, dichotomized as not well‐suited/uncertain versus well‐suited. Prospective associations were tested with multivariable logistic regression using April covariates. As a secondary outcome, 6‐month transitions—deterioration (well‐suited to not/uncertain) and improvement (not/uncertain to well‐suited)—were compared by school year with McNemar's tests.

**Results:**

First‐year students showed net deterioration, whereas third‐year students showed relative stability/improvement. McNemar's tests indicated net deterioration in first‐year students (4 vs. 26; *p* < 0.001) and second‐year students (5 vs. 17; *p* = 0.019), but not in third‐year students (13 vs. 7; *p* = 0.263). In the prospective model, higher baseline positive self‐compassion was associated with lower odds of being not well‐suited/uncertain at 6 months (OR = 0.90 [0.83–0.96]; *p *= 0.003), whereas higher HADS‐D was associated with higher odds of being not well‐suited/uncertain at 6 months (OR = 1.11 [1.00–1.22]; *p* = 0.044).

**Conclusions:**

Lower self‐compassion and higher depressive symptoms at baseline were associated with later reports of being not well‐suited/uncertain. These findings indicate associations between self‐compassion and perceived suitability; causal inferences cannot be made from this observational study.

## 1 Introduction

Professional identity formation (PIF) is commonly described as the developmental process through which learners transition from laypersons to professionals [1]. Core professional values are shaped through everyday clinical experience—one of the central engines of identity development [2]. Perceived suitability for the profession can be conceptualized as a proximal self‐appraisal of fit and emerging commitment, capturing how strongly an individual experiences congruence between the self and the professional role [3]. While perceived suitability does not exhaust the construct of PIF, it can serve as an accessible indicator of learners' ongoing identity‐related appraisal within the training context. In many dental hygiene programs in Japan, substantive clinical placements begin in the third year; thus, identity‐focused learning objectives are typically emphasized from that stage onward. In contrast, the first year is largely didactic. During this early phase, student‐reported indicator—perceived professional suitability (i.e., whether one feels well‐suited to the profession)—is likely to influence study motivation and day‐to‐day performance. However, empirical work describing perceived suitability among dental hygiene students and its correlates remains limited.

Our group has examined psychological correlations in this population. Subjective health perception was positively associated with innate resilience and negatively associated with stress responses [4]. More recently, we reported that uneasiness about one's professional future was associated with higher scores on the negative facet of self‐compassion among dental hygiene students, independently with their resilience [5]. Self‐compassion—framed in both Buddhist and Western psychological traditions—entails self‐kindness, recognition of common humanity, and mindful balance, as opposed to self‐judgment, isolation, and overidentification [6, 7]. These constructions are one of the plausible determinants of how students appraise their fit for a profession during training.

Against this background, the present longitudinal study followed dental hygiene students from the start of the academic term (April) to 6 months (September) to examine factors related to changes in perceived suitability as a dental hygienist. Self‐compassion may be relevant to PIF because student self‐compassion may play some role in the degree of discrepancy between the ideal professional image and student self‐concept [8]. Thus, we focused on self‐compassion (positive and negative) as a primary psychological factor—given its prior association with professional unease [5]—and explored its prospective relationship with later perceptions of being not well‐suited or uncertain, alongside measures of perceived stress and anxiety/depressive symptoms.

## 2 Methods

### 2.1 Study Design and Participants

We conducted a longitudinal questionnaire survey between April and September 2025. The target population consisted of 450 students enrolled in four dental hygiene schools in Fukuoka Prefecture, Japan. Students were eligible if they completed the questionnaires at both time points. Those on a leave of absence due to psychological conditions or mental health concerns were excluded.

Students progressed through a 3‐year program. In Year 1, instruction is classroom and laboratory based with no clinical placement and no patient contact. In Year 2, students supplement didactic coursework with basic clinical skills training (including peer‐to‐peer practice in caries prevention, scaling, and periodontal probing) but still have no formal clinical placements or patient contact. In Year 3, students complete 900 h of supervised clinical placement (mandated by national regulation) between April and September, at the university hospital, affiliated hospitals, and community/long‐term‐care sites, providing direct patient care (preventive services, perioperative oral care, dysphagia screening/education, and interprofessional case management).

Ethical approval was obtained from the Medical Ethics Committee of Kyushu Dental University (No. 25–1). Because the study involved only self‐administered questionnaires and presented minimal risk, it was implemented under an opt‐out framework in accordance with the Ethical Guidelines for Medical and Biological Research Involving Human Subjects in Japan. Study information was disclosed publicly, and students were given the opportunity to decline participation; written informed consent was, therefore, not required.

### 2.2 Data Collection

Surveys were administered electronically using Microsoft Forms, with all questions set as mandatory to prevent missing data. Each survey required approximately 5 min to complete.

All responses were collected and managed in an anonymized manner. Personally identifiable information such as names or student number was not obtained. Each student was assigned a unique identification code to link pre‐ and post‐survey data while preserving confidentiality. Participation was voluntary, and students could withdraw from the study without any consequences for their academic record.

### 2.3 Sample Size Calculation

An a priori power analysis for logistic regression was performed using G*Power (version 3.1.9.7; two‐sided). We assumed an odds ratio (OR) of 1.50, a base event rate under the null hypothesis of Pr(*Y* = 1|*X* = 1) = 0.50, *α* = 0.05, and power (1–*β*) = 0.80. To account for the inclusion of multiple predictors in the logistic regression model, we specified the expected proportion of variance in the predictor of interest explained by the remaining covariates (R² for other predictors) as 0.10, following the procedure implemented in G*Power. Predictor distributions were specified according to their type (continuous variables treated as standardized normal, categorical variables as binomial). The required sample size was calculated as 231. Allowing for an expected response rate of 70% and a dropout rate of 20%, the estimated number of recruits needed was 413. Accordingly, all 450 students enrolled in the dental hygiene schools in April were included in the study with the cooperation of the schools, thereby ensuring adequate statistical power.

### 2.4 Questionnaire

To evaluate self‐compassion, we employed the short form of the Japanese version of the Self‐Compassion Scale (SCS) [5, 7, 9]. The instrument comprises 12 items, each rated on a five‐point Likert scale. The SCS is divided into two subdomains: positive SCS and negative SCS. The positive subscale includes six items assessing self‐kindness, common humanity, and mindfulness, whereas the negative subscale contains six items reflecting self‐judgment, isolation, and overidentification. Subscale scores range from 6 to 30. The total SCS score was calculated by adding the positive SCS score to the difference between 36 and the negative SCS score [total SCS = positive SCS + (36 – negative SCS)], resulting in a possible total range of 12–60. Higher scores on the positive SCS and total SCS indicate greater self‐compassion, whereas higher scores on the negative scale SCS reflect lower levels of self‐compassion.

Perceived stress was assessed with the Perceived Stress Scale (PSS) [10], which measures the degree to which life circumstances are appraised as stressful. The PSS has been described as an appropriate tool for capturing outcomes of stress based on person–environment interactions [11]. The 10‐item version consists of six negatively worded and four positively worded questions, with total scores ranging from 0 to 40. Higher scores indicate greater perceived stress.

Emotional status was assessed using the Hospital Anxiety and Depression Scale (HADS) [12, 13]. This scale includes two subdomains—anxiety (HADS‐A) and depression (HADS‐D)—each consisting of seven items rated on a four‐point scale (0–3). Subscale scores of ≤ 7 indicate no clinically relevant anxiety or depression, scores of 8–10 suggest borderline cases, and scores of ≥ 11 are indicative of probable caseness. A validated Japanese translation was used [13]. Higher scores reflect more severe anxiety or depressive symptoms.

The internal consistency of the measures was acceptable. Cronbach's *α* values were 0.759 for positive SCS, 0.855 for negative SCS, 0.728 for total SCS, 0.757 for PSS, 0.808 for HADS‐A, 0.686 for HADS‐D, and 0.835 for HADS‐total.

Demographic characteristics (gender and school year) were also recorded. Perceived suitability for becoming a dental hygienist was assessed using three options: well‐suited, neither, or not well‐suited. The perceived burden of clinical/practical training was evaluated with a binary response (yes or no). Self‐rated health status was reported on a five‐point scale (very healthy, healthy, neither, not very healthy, not healthy), which was subsequently collapsed into three categories (very healthy, healthy, and other) for statistical analyses.

### 2.5 Outcome

The primary outcome was the perception of being not well‐suited to work as a dental hygienist at the 6‐month follow‐up (September 2025). Students chose from three options (well‐suited, not well‐suited, or neither). For analysis, not well‐suited and neither responses were coded as 1, and well‐suited as 0. Thus, an OR greater than 1 indicates a higher likelihood of perceiving oneself as not well‐suited (including uncertain responses).

The secondary outcome was the change in perceived suitability between baseline (April 2025) and 6 months (September 2025). Responses at the two time points were cross‐classified into four transition categories:

- not well‐suited/uncertain → not well‐suited/uncertain (stable negative),
- not well‐suited/uncertain → well‐suited (improvement),
- well‐suited → not well‐suited/uncertain (deterioration), and
- well‐suited → well‐suited (stable positive).

### 2.6 Missing Data and Weighting

To address attrition, we used stabilized inverse probability weighting (IPW). For each student *i*, the probability of being observed at follow‐up was estimated by logistic regression using baseline covariates, denoted *p̂*_i_ = Pr(follow‐up_i_ = 1|*X*_i_). Stabilized weights were defined as *w*_i_ = *p̄*/*p̂*_i_, where *p̄* is the overall follow‐up rate (0.729). Weight diagnostics indicated a well‐behaved distribution (mean 1.021, SD 0.159, range 0.79–1.85); therefore, no trimming was applied. Primary models were re‐estimated with IPW and reported alongside complete‐case estimates.

### 2.7 Perceived Professional Suitability

At 6 months, students answered a single global item: “Do you feel you are well‐suited to be a dental hygienist?” (well‐suited/neither/not well‐suited). Because this is a single‐item measure, internal consistency (Cronbach's *α*) is not applicable, and test–retest data were not collected for this item. In the primary analysis, we dichotomized the outcome as not well‐suited (= 1; pooling “neither” with “not well‐suited”) versus well‐suited (= 0) to operationalize any deviation from clear professional fit. To address the potential distinctiveness of “neither,” we conducted sensitivity analyses using a three‐category multinomial model (well‐suited/neither/not well‐suited).

### 2.8 Statistical Analysis

Descriptive statistics were calculated for baseline characteristics. Between‐group differences in categorical variables were examined using Pearson's chi‐square test, and effect sizes were expressed as *φ* or Cramér's *V*. For continuous variables, group comparisons were performed with the Mann–Whitney *U* test, and effect sizes were expressed as *r*.

Changes in perceived suitability from April to September were cross‐tabulated, and McNemar's test was used to compare the frequency of improvement versus deterioration within each academic year. Adjusted standardized residuals (ASRs) were also examined to identify cells contributing disproportionately to chi‐square values. Effect sizes were summarized using ORs, 95% confidence intervals (CIs), and Cohen's *g*.

For within‐subject changes in continuous variables such as SCS, PSS, and HADS between April and September, the Wilcoxon signed‐rank test was applied, and effect sizes were expressed as *r*.

Binary logistic regression was applied to identify baseline factors associated with perceiving oneself as not well‐suited/uncertain at 6 months. Independent variables included self‐compassion (positive and negative), perceived stress (PSS), emotional status (HADS‐A, HADS‐D), health status, and academic year. The coding of the dependent variable was 1 = not well‐suited/uncertain, 0 = well‐suited. ORs and 95% CIs were derived from regression coefficients (*B*), and multicollinearity was assessed using variance inflation factors (VIF).

Model fit was evaluated using the Hosmer–Lemeshow goodness‐of‐fit test, explanatory power by Nagelkerke's *R* ^2^, and discriminative ability by the area under the receiver operating characteristic (ROC) curve (AUC).

A two‐tailed significance level of *p* < 0.05 was considered statistically significant. All analyses were conducted using SPSS Statistics, version 28.0.1.0 (IBM Corp., Armonk, NY, USA).

## 3 Results

Of 450 eligible students, 402 students participated at baseline, 293 completed both surveys, and 109 were dropouts. Baseline characteristics were broadly similar between completers and dropouts, except for student year (Table 1). Because females constituted the vast majority of participants, sex‐stratified analyses were not conducted. Among completers, the distribution across years was approximately even (about one‐third per year), whereas dropouts were more common among first‐year students; however, the effect size was small.

**TABLE 1:** Baseline characteristics of participants (completers vs. dropouts).

	Completers (*N* = 293)	Dropouts (*N* = 109)	*p* value	Effect size
Sex, *n*				
Male	2 (0.7)	1 (0.9)	0.252	0.083
Female	291 (99.3)	107 (98.2)		
Other	0 (0)	1 (0.9)		
Student year, *n*				
First	94 (32.1)	55 (50.5)	0.003	0.169
Second	101 (34.5)	27 (24.8)		
Third	98 (33.4)	27 (24.8)		
Suitability for being a dental hygienist, *n*				
Not well‐suited	159 (54.3)	55 (50.5)	0.497	0.034
Well‐suited	134 (45.7)	54 (49.5)		
Perceived burden of clinical/practical training, *n*				
Yes	180 (61.4)	58 (53.2)	0.136	0.074
No	113 (38.6)	51 (46.8)		
Health status, *n*				
Very healthy	105 (35.8)	38 (34.9)	0.894	0.024
Healthy	149 (50.9)	58 (53.2)		
Other	39 (13.3)	13 (11.9)		
Self‐compassion, *m*				
Positive SCS	19 [17–22]	19 [16–21]	0.869	0.01
Negative SCS	19 [15–22]	19 [15–22]	0.899	0.01
Total SCS	36 [33–40]	36 [32–40]	0.758	0.02
PSS, *m*	20 [15–24]	19 [14–24.5]	0.728	0.02
HADS, *m*				
HADS‐A	6 [4–10]	5 [3–9]	0.057	0.09
HADS‐D	5 [3–9]	5 [3–7]	0.583	0.03
HADS‐total	12 [7.5–18]	11 [7–16]	0.135	0.07

*Note*: Values are presented as number (*n*) with % for categorical variables and median (*m*) [interquartile range] for continuous variables. For descriptive purposes in Table 1, “neither” was grouped with “not well‐suited.” Effect size is expressed as Cramér's *V* (or *φ* for 2 × 2 tables) for categorical variables and *r* for continuous variables.

Abbreviations: HADS, hospital anxiety and depression scale; PSS, perceived stress scale; SCS, self‐compassion scale.

Attrition at follow‐up was 27.1% (109/402). To assess the impact of missingness, we re‐estimated the primary models using stabilized IPW based on baseline covariates. IPW‐weighted estimates were closely aligned with complete‐case estimates (Table S1): both the direction and the magnitude of associations were materially unchanged, and CIs overlapped substantially. For example, the adjusted OR for the higher‐burden group remained similar with complete‐case versus IPW (OR 1.73, 95% CI 1.06–2.81 vs. OR 1.62, 95% CI 1.01–2.62). Associations for baseline positive and negative self‐compassion were virtually identical under both specifications (positive: OR 0.89; negative: OR 1.06). Taken together, these findings indicate that the study conclusions are robust to the handling of missing data.

Median changes of psychological scales (SCS, PSS, and HADS) from baseline to 6 months were generally small. Notable improvements were observed in first‐year students for HADS‐A (6 [3.8–11] to 5.5 [3–9.3], *p* = 0.004) and HADS‐total (12 [7–18] to 10 [6–15.3], *p* = 0.025) (Table 2). Although both positive SCS and negative SCS showed statistically significant decreases in the third‐year students, their effect sizes (0.02) indicated negligible practical change.

**TABLE 2:** Within student year changes in psychological measures from baseline to 6 months.

	Baseline	After 6 months	*p* value	*r*
First				
Positive SCS	19 [16.8–21.3]	19 [16.8–21.3]	0.623	0.05
Negative SCS	19 [15–23]	19 [14–22]	0.173	0.14
Total SCS	36 [32–40]	36 [32.8–40.3]	0.557	0.06
PSS	18.5 [13–23.3]	20 [16–24]	0.229	0.12
HADS‐A	6 [3.8–11]	5.5 [3–9.3]	0.004	0.29
HADS‐D	5 [3–8]	5 [3–7]	0.410	0.08
HADS‐total	12 [7–18]	10 [6–15.3]	0.025	0.23
Second				
Positive SCS	19 [17–22]	18 [17–22]	0.752	0
Negative SCS	19 [15–22]	18 [14.5–22]	0.157	0.01
Total SCS	36 [33–40.5]	36 [32.5–40.5]	0.568	0.01
PSS	19 [15.5–25]	18 [13.5–23]	0.053	0.02
HADS‐A	5 [3–10]	6 [3–9]	0.491	0.01
HADS‐D	5 [3–7.5]	5 [3–8]	0.661	0
HADS‐total	11 [6.5–16]	11 [7–16.5]	0.929	0
Third				
Positive SCS	19 [17–22]	19 [16–21]	0.036	0.02
Negative SCS	19 [15–22]	18 [13–22]	0.020	0.02
Total SCS	36 [33–40]	36 [33–41]	0.763	0
PSS	20.5 [16–24]	19 [15–23]	0.073	0.02
HADS‐A	8 [4–11]	7 [3–11]	0.141	0.02
HADS‐D	6 [4–10]	6 [3–9.3]	0.194	0.01
HADS‐total	14 [9–20]	12 [6.8–19]	0.105	0.02

*Notes*: Values are presented as number (*n*) with % for categorical variables and median (*m*) [interquartile range] for continuous variables. Wilcoxon signed‐rank test; effect size *r* = |*Z*|/$\sqrt{N}.$

Abbreviations: HADS, hospital anxiety and depression scale; PSS, perceived stress scale; SCS, self‐compassion scale.

Transitions in perceived suitability over 6 months differed by student year (Table 3). Transitions were summarized as deterioration (well‐suited→not well‐suited) and improvement (not well‐suited→well‐suited). In first‐year students, deterioration transitions occurred more often than expected (ASR = 3.3), whereas not well‐suited to not well‐suited occurred less often (ASR = − 3.2). In contrast, third‐year students showed fewer deterioration transitions (ASR = − 3.2) and more improvement (ASR = 2.7). Paired (McNemar) tests indicated net deterioration in first‐year students (improvement = 4 vs. deterioration = 26; *p* < 0.001; *g* = − 0.234; OR = 0.14, 95% CI 0.05–0.44) and in second‐year students (improvement = 5 vs. deterioration = 17; *p* = 0.019; *g* = − 0.119; OR = 0.24, 95% CI 0.11–0.80), but not in third‐year students (improvement = 13 vs. deterioration = 7; *p* = 0.263; *g* = 0.061; OR = 1.86, 95% CI 0.74–4.66). Overall, deterioration exceeded improvement (improvement = 22 vs. deterioration = 50; *p* = 0.002; *g* = − 0.096; OR = 0.44, 95% CI 0.27–0.72) (Table 4). No statistically significant change in the perceived burden of clinical/practical training was observed over the 6‐month period (Table S2).

**TABLE 3:** Transitions in perceived suitability for being a dental hygienist, with adjusted standardized residuals.

Student year		Not well‐suited ↓ Not well‐suited	Not well‐suited ↓ Well‐suited	Well‐suited ↓ Not well‐suited	Well‐suited ↓ Well‐suited
First	*n* (%)	31 (33.0)	4 (4.3)	26 (27.7)	33 (35.1)
	ASR	−3.2	−1.5	3.3	1.7
Second	*n* (%)	58 (57.4)	5 (5.0)	17 (16.8)	21 (20.8)
	ASR	2.7	−1.2	−0.1	−2.2
Third	*n* (%)	48 (49.0)	13 (13.3)	7 (7.1)	30 (30.6)
	ASR	0.5	2.7	−3.2	0.5

*Note*: Cells with |ASR| ≥ 1.96 were considered significant contributors to the chi‐square.

Abbreviation: ASR, adjusted standardized residual.

**TABLE 4:** Paired changes in perceived suitability for being a dental hygienist.

Student year	Not well‐suited ↓ Well‐suited (improvement)	Well‐suited ↓ Not well‐suited (deterioration)	*p* value	Cohen's *g*	OR (95% CI)
First	4	26	< 0.001	−0.234	0.14 (0.05–0.44)
Second	5	17	0.019	−0.119	0.24 (0.11–0.80)
Third	13	7	0.263	0.061	1.86 (0.74–4.66)
Overall	22	50	0.002	−0.096	0.44 (0.27–0.72)

*Note*: McNemar's test. Cohen's *g* = (improvement − deterioration) / *N*; OR = improvement / deterioration.

Abbreviations: CI, confidence interval; OR, odds ratio.

In the multivariable model using April covariates (primary analysis), higher positive SCS was associated with lower odds of being not well‐suited/uncertain at 6 months (OR = 0.90, 95% CI 0.83–0.96; *p* = 0.003), whereas higher baseline HADS‐D was associated with higher odds of being not well‐suited/uncertain at 6 months (OR = 1.11, 95% CI 1.00–1.22; *p* = 0.044) (Table 5). Model calibration and discrimination were acceptable (Hosmer–Lemeshow *χ* ^2^ = 7.96, *p *= 0.438; Nagelkerke *R* ^2^ = 0.171; AUC = 0.72, 95% CI 0.66–0.78). Multicollinearity was negligible (all VIF < 5).

**TABLE 5:** Baseline factors associated with perceiving oneself as not well‐suited to be a dental hygienist at 6 months.

	*B* (SE)	OR (95% CI)	*p* value	VIF
Pre‐positive SCS	−0.11 (0.04)	0.90 (0.83–0.96)	0.003	1.09
Pre‐negative SCS	0.05 (0.03)	1.05 (0.98–1.12)	0.139	1.63
Pre‐PSS	0.01 (0.03)	1.01 (0.96–1.07)	0.650	1.95
Pre‐HADS‐A	−0.06 (0.04)	0.94 (0.87–1.03)	0.186	2.21
Pre‐HADS‐D	0.10 (0.05)	1.11 (1.00–1.22)	0.044	1.70
Student year				1.16
First	−0.61 (0.34)	0.55 (0.28–1.07)	0.077	—
Second	0.41 (0.34)	1.51 (0.77–2.94)	0.229	—
Third	Reference			—
Perceived burden of clinical/practical training (pre)				1.28
Yes	0.80 (0.31)	2.22 (1.22–4.00)	0.009	—
No	Reference			

*Note*: Coefficients are shown as *B* (SE). “pre‐” means data assessed at baseline. Model means multivariable logistic regression with not well‐suited as the outcome (1 = not well‐suited, 0 = well‐suited). OR = exp(*B*); 95% CI by Wald method; VIF < 5. Hosmer–Lemeshow test = 7.96 (*p* = 0.438). Nagelkerke *R* ^2^ = 0.171. Area under the receiver operating characteristic curve = 0.72 (95% CI 0.66–0.78), *p* = 0.031.

Abbreviations: *B*, partial regression coefficient; CI, confidence interval; OR, odds ratio; SE, standard error; VIF, variance inflation factor.

For context, the cross‐sectional model using September covariates showed a similar association with lower odds for positive SCS (OR = 0.91, 95% CI 0.85–0.97; *p* = 0.005), whereas higher self‐compassion (negative) (OR = 1.07, 95% CI 1.01–1.14; *p* = 0.030), perceived training burden (OR = 2.31, 95% CI 1.31–4.07; *p* = 0.004), and first‐year status (OR = 2.44, 95% CI 1.28–4.63; *p* = 0.007) were associated with being not well‐suited (Table S3). Model performance was similar (Hosmer–Lemeshow χ^2^ = 7.59, *p* = 0.475; Nagelkerke R^2^ = 0.179; AUC = 0.73, 95% CI 0.67–0.78). As sensitivity analyses, we performed multinomial models (“well‐suited”, “neither”, and “not well‐suited”). In these models (reference = well‐suited), higher pre‐positive self‐compassion was associated with lower odds of both “neither” and “not well‐suited”, while second‐year status and perceived burden were associated with higher odds of “not well‐suited” (Table S4). Together with the complete‐case/IPW comparisons (Table S1), the multinomial results are consistent with Table 5, supporting the robustness of our findings.

## 4 Discussion

In this longitudinal study of dental hygiene students in Japan, approximately half of the participants perceived themselves as either not well‐suited or uncertain about their suitability as a dental hygienist at the end of first term. Negative or uncertain perceptions were particularly common among first‐year students, many of whom shifted from initially feeling well‐suited to reporting uncertainty or lack of suitability at follow‐up. McNemar's tests indicated a significant net deterioration in first‐ and second‐year students, whereas third‐year students showed no statistically significant change. These stage‐specific demands—namely, a largely didactic curriculum in first year and the onset of basic clinical exposure in the second year—may partly account for the deterioration observed in both years. One possible explanation for this pattern, consistent with PIF theory, is that broader and more authentic clinical exposure in the third year—through placements in both private dental practices and university hospitals—may increase opportunities for role modeling and legitimate participation, thereby supporting the consolidation of professional understanding and stabilizing perceived suitability. By contrast, the early training years may be characterized by heightened uncertainty, academic stress, and fewer opportunities for authentic role enactment, which could undermine perceived suitability before students have sustained clinical experiences. Most recently, a qualitative study identified six themes shaping PIF among Japanese dental hygiene students [14]: behavioral characteristics of role models, stimulation from clinical situations, efforts to adapt to clinical practice, social context in dental practice, relationships between dental hygienists and others, and the fellowship of learning together. Building on these findings, the authors also suggested that interprofessional education may facilitate students' understanding and professional cognition [15].

Prospective analyses indicated that higher baseline positive SCS was associated with lower odds of later perceiving oneself as not well‐suited/uncertain, whereas higher baseline depressive symptoms (HADS‐D) were associated with higher odds of being not well‐suited/uncertain.

### 4.1 Implications for Education

The pattern of deterioration in first‐year students underscores the need for early educational support. Orientation programs and structured mentoring during the initial phase of training may help students adapt to academic and clinical demands and mitigate loss of confidence. The observed association between higher positive self‐compassion and lower odds of being not well‐suited/uncertain suggests that fostering self‐kindness, mindfulness, and resilience could help maintain a positive professional identity; integrating reflective practice, mindfulness, or self‐compassion exercises into the curriculum may therefore support students' psychological well‐being. Previous narrative review demonstrated that mindfulness meditation reduced anxiety in 82% of studies and stress in 74% of studies [16]. Consistent with this, our prior work showed that self‐compassion is lower in students expressing concerns about their professional future, and that the negative facet of self‐compassion is independently associated with resilience [5]. In addition, the association between depressive symptoms and unfavorable perceptions underscores the potential importance of mental‐health screening and timely support within training institutions. Establishing clear referral pathways and offering stress‐management resources may help prevent disengagement from the professional trajectory.

### 4.2 Limitations

This study has several limitations. First, causal inference is not possible from these observational data; all results should be interpreted as associations. Despite adjustment for measured covariates (and use of sensitivity analyses where applicable), residual and unmeasured confounding, reverse causation, selection processes (e.g., attrition), and measurement/misclassification error may bias the observed associations. Temporal ordering cannot be established with certainty for all variables; therefore, the findings should not be interpreted as causal effects. Future randomized or quasi‐experimental studies will be required to test causal hypotheses. Second, the study was conducted in four schools within a single prefecture, which may limit generalizability. Moreover, the cohort consisted predominantly of female dental hygiene students within a single national context, which may limit generalizability to other curricula, regions, and more gender‐balanced student bodies. Third, despite adjustment for key psychological and demographic covariates, unmeasured confounding (e.g., academic performance, clinical evaluation, and social support) may remain, and causal inferences cannot be drawn from these observational data. Fourth, potential selection and attrition bias cannot be excluded under the opt‐out framework and given the observed dropouts. Fifth, the follow‐up period was 6 months; longer trajectories of professional identity development were not captured. Sixth, given 27.1% attrition (109/402), selection bias is possible. IPW produced estimates similar to complete‐case models, with slight attenuation, implying that any attrition‐related bias is likely small; however, residual bias from unmeasured dropout predictors may remain. Seventh, the outcome (the perception of being not well‐suited to dental hygienist) was captured by a single item, with no test–retest reliability data, which may introduce non‐differential measurement error and potential misclassification, particularly regarding the “neither” category under dichotomization. Such error would generally attenuate associations toward the null. We therefore report multiple codings and sensitivity analyses to demonstrate robustness (Table S4).

### 4.3 Implications for Educational Interventions to Maintain Perceived Suitability for the Dental Hygiene Profession

Evidence from allied health education indicates that brief, embedded psychological skills training can stabilize students' perception of professional fit. Mindfulness programs for medical trainees yield small‐to‐moderate reductions in stress, with stronger effects when time demands are modest—supporting short in‐class routines rather than large standalone courses [17]. Compassion/self‐compassion training in nursing students lowers stress and anxiety, plausibly via gains in self‐compassion, and can be delivered effectively online or within regular class time [18]. Near‐peer mentoring also has a demonstrated role: theory‐informed work shows it scaffolds PIF through communities of practice and structured reflection, helping sustain a stable sense of professional suitability [19].

Per our findings, it may be useful to consider year‐specific entry points for brief self‐compassion–oriented support. In Year 1, students are mainly exposed to didactic‐based foundational and professional subjects that differ markedly from their high school curriculum; at this stage, those with lower positive self‐compassion may be especially likely to experience the novelty and difficulty of the content as evidence that they are “not suited” to the profession. By contrast, 6 months into Year 2, students are beginning preclinical skills training (e.g., scaling, probing, and other fine motor procedures), and some may interpret early technical failures as a sign of personal inadequacy rather than as a normal part of skill acquisition. In Year 3, roughly 6 months after the start of extended clinical placements, students are working with real patients in authentic practice settings; here, practical setbacks, communication difficulties, and emotionally demanding encounters may all fuel doubts about their suitability, particularly among those with low positive self‐compassion.

Across all 3 years, our results suggest that students with lower positive self‐compassion may warrant targeted, low‐burden interventions embedded in existing teaching. One feasible approach would be to integrate brief mindfulness‐informed exercises (e.g., a short centering or breathing practice, guided non‐judgmental observation of thoughts and feelings about recent academic or clinical challenges) into selected classes or clinical conferences. Another is to use structured reflective writing tasks in which students describe a recent difficulty and then generate self‐directed comments explicitly mapped to the three domains of self‐compassion (self‐kindness vs. self‐judgment, common humanity vs. isolation, and balanced awareness vs. over‐identification). Such exercises might help students normalize struggles, respond more kindly to themselves, and maintain a sense of professional suitability, while allowing teachers to focus additional support on those who screen low for positive self‐compassion at the beginning of each year.

#### 4.3.1 Implementation Barriers and Solutions

*Time pressure in a crowded curriculum*: Integrate 5–10‐min micro‐doses into existing lectures/clinics; use ready‐to‐run scripts and a consistent opening routine (lighter‐touch formats are favored in mindfulness meta‐analytic evidence) [17].

*Stigma around help‐seeking*: Make practices universal (whole‐class delivery), keep debriefs non‐evaluative and separate from grading, and provide optional digital audio guides from the compassion module [18].

*Faculty bandwidth and variable confidence*: Offer a brief train‐the‐trainer (two 90‐min sessions per year), distribute standardized scripts for mindfulness/self‐compassion exercises, and leverage near‐peer mentors to diffuse delivery [19].

## 5 Conclusion

In this longitudinal cohort of Japanese dental hygiene students, approximately half reported being not well‐suited or uncertain about their suitability at 6 months. Year‐stratified analyses showed a net deterioration from April to September in first‐ and second‐year students, with no significant change in third‐year students. In prospective models, higher baseline positive self‐compassion was associated with lower odds of later perceiving oneself as not well‐suited/uncertain, whereas higher baseline depressive symptoms (HADS‐D) were associated with higher odds. Cross‐sectional analyses at 6 months were consistent with the association between higher self‐compassion and lower odds of being not well‐suited/uncertain, and additionally, perceived training burden and first‐year status were correlates of being not well‐suited/uncertain. Together, these results delineate year‐related patterns and psychological factors linked to students' perceived professional suitability over a 6‐month period. These findings should be interpreted as associations rather than causal effects. Future research should examine these associations over longer periods and across institutions, incorporate qualitative data to capture identity narratives, and employ designs that strengthen causal inference (e.g., quasi‐experimental approaches or interventions that cultivate self‐compassion) to evaluate whether changes in self‐compassion precede changes in perceived suitability.

## Author Contributions

Maya Izumi contributed to the conception and design of this study, data curation, and drafted original manuscript. Hsiu‐Yueh Liu contributed to the formal analysis and edited the final manuscript. Sumio Akifusa contributed to the conceptualization and design of this study, data analysis, drafted the final manuscript, and developed the tables. All authors agree and are accountable for all aspects of the work and have given their approval for this publication.

## Supporting information




**Supporting Figure 1**: jdd70174‐sup‐0001‐tableS1‐S4.docx
