# Supplementary material for: Perceived Suitability for Being a Dental Hygienist and Self‐Compassion: A 6‐Month Longitudinal Cohort Study
Source: J Dent Educ. 2026 Feb 4;90(10):1525–32. doi: 10.1002/jdd.70174 (PMC13665589; doi:10.1002/jdd.70174)
Supplement: Supplementary file 1 [file JDD-90-1525-s001.docx]

Supplemental Table S1 Comparison of estimates under complete-case analysis and stabilized inverse probability weighting

| variables | Complete-case OR [95%CI] | IPW OR [95%CI] |
| --- | --- | --- |
| pre-positive SCS | 0.888 [0.837-0.943] | 0.888 [0.838-0.942] |
| pre-negative SCS | 1.057 [1.002-1.115] | 1.057 [1.003-1.115] |
| pre-PSS | 1.010 [0.965-1.056] | 1.011 [0.944-1.082] |
| pre-HADS-A | 0.989 [0.924-1.059] | 0.990 [0.924-1.059] |
| pre-HADS-D | 1.082 [0.998-1.172] | 1.078 [0.994-1.168] |
| Student year |  |  |
| 1st | 0.874 [0.503-1.520] | 0.869 [0.490-1.541] |
| 2nd | 2.498 [1.425-4.378] | 2.556 [1.468-4.451] |
| 3rd | reference |  |
| Perceived burden of clinical/practical training (pre) | | |
| Yes | 1.725 [1.060–2.808] | 1.624 [1.007–2.619] |
| No | reference |  |

IPW: inverse probability weighting

Adjusted for listed covariates. IPW used stabilized weights 𝑝 = 0.729 with no trimming (mean 1.021; SD 0.159; range 0.79–1.85). Complete-case models used listwise deletion. Estimates were similar across approaches, indicating robustness to attrition handling.

Supplemental Table S2. Paired change in perceived burden of clinical/practical training

|  |  |  | After 6 months | |  |
| --- | --- | --- | --- | --- | --- |
|  |  |  | Yes | No | p value |
| Baseline | 1st | Yes | 29 (82.9) | 6 (17.1) | 0.078 |
|  |  | No | 15 (25.4) | 44 (74.6) |  |
|  | 2nd | Yes | 57 (82.6) | 12 (17.4) | 0.362 |
|  |  | No | 18 (56.3) | 14 (43.8) |  |
|  | 3rd | Yes | 66 (86.8) | 10 (13.2) | 0.629 |
|  |  | No | 7 (31.8) | 15 (68.2) |  |

McNemar test

Supplemental Table S3. Factors at 6 months associated with perceiving oneself as not well-suited to be a dental hygienist

|  | B (SE) | OR (95% CI) | p value | VIF |
| --- | --- | --- | --- | --- |
| post-positive SCS | −0.10 (0.04) | 0.91 (0.85–0.97) | 0.005 | 1.07 |
| post-negative SCS | 0.07 (0.03) | 1.07 (1.01–1.14) | 0.030 | 1.49 |
| post-PSS | 0.04 (0.03) | 1.04 (0.99–1.10) | 0.111 | 1.63 |
| post-HADS-A | −0.07 (0.04) | 0.93 (0.86–1.01) | 0.094 | 2.03 |
| post-HADS-D | 0.04 (0.05) | 1.04 (0.95–1.15) | 0.386 | 1.74 |
| Student year |  |  |  | 1.12 |
| 1st | 0.89 (0.33) | 2.44 (1.28–4.63) | 0.007 | — |
| 2nd | 0.37 (0.33) | 1.44 (0.75–2.78) | 0.273 | — |
| 3rd | reference |  |  | — |
| Perceived burden of clinical/practical training (post) | | | | 1.13 |
| Yes | 0.84 (0.29) | 2.31 (1.31–4.07) | 0.004 | — |
| No | reference |  |  |  |

Coefficients are shown as B (SE)

B: partial regression coefficient, SE: standard error, OR: odds ratio, CI: confidence interval

VIF: variance inflation factor

“post-“ means data assessed after 6 months.

Model: multivariable logistic regression with not well-suited at 6 months as the outcome (1 = not well-suited, 0 = well-suited). OR = exp(B); 95% CI by Wald method; VIF < 5.

Hosmer–Lemeshow test: 7.59 (p = 0.475)

Nagelkerke R^2^ = 0.179

Area under the Receiver Operating Characteristic curve = 0.73 (95% CI 0.67–0.78), p = 0.030

Supplemental Table S4 Sensitivity analysis: three-category multinomial logistic regression of perceived professional suitability at 6 months (reference = well-suited).

A. Neither

|  | B (SE) | OR (95% CI) | p value |
| --- | --- | --- | --- |
| post-positive SCS | −0.08 (0.04) | 0.92 (0.85-1.00) | 0.040 |
| post-negative SCS | 0.05 (0.04) | 1.05 (0.98-1.13) | 0.152 |
| pre-PSS | −0.01 (0.03) | 0.99 (0.94-1.05) | 0.800 |
| pre-HADS-A | −0.05 (0.05) | 0.95 (0.87-1.04) | 0.279 |
| pre-HADS-D | 0.10 (0.05) | 1.11 (1.00-1.23) | 0.056 |
| Student year | |  |  |
| 1st | 0.47 (0.37) | 1.60 (0.78-3.28) | 0.201 |
| 2nd | 0.77 (0.36) | 2.17 (1.07-4.39) | 0.032 |
| 3rd | reference |  |  |
| Perceived burden of clinical/practical training (pre) | | | |
| Yes | 0.60 (0.33) | 1.82 (0.96-3.46) | 0.068 |
| No | reference |  |  |

B. Not well-suited

|  | B (SE) | OR (95% CI) | p value |
| --- | --- | --- | --- |
| post-positive SCS | −0.17 (0.05) | 0.84 (0.77-0.92) | <0.001 |
| post-negative SCS | 0.05 (0.04) | 1.05 (0.97-1.14) | 0.238 |
| pre-PSS | 0.05 (0.04) | 1.05 (0.98-1.13) | 0.141 |
| pre-HADS-A | −0.08 (0.06) | 0.93 (0.83-1.03) | 0.167 |
| pre-HADS-D | 0.10 (0.06) | 1.10 (0.98-1.24) | 0.110 |
| Student year | |  |  |
| 1st | 0.86 (0.46) | 2.35 (0.96-5.74) | 0.061 |
| 2nd | 1.46 (0.42) | 4.29 (1.87-9.81) | <0.001 |
| 3rd | reference |  |  |
| Perceived burden of clinical/practical training (pre) | | | |
| Yes | 1.21 (0.40) | 3.36 (1.52-7.40) | 0.003 |
| No | reference |  |  |

Outcome categories: well-suited (ref), neither, not well-suited. Values are OR (95% CI); predictors assessed at baseline; “pre–” indicates baseline.)
